# AI-driven magnetoencephalography biomarkers in dementia risk prediction: current evidence, challenges and future perspectives

**DOI:** 10.3389/frdem.2026.1743627

**Published:** 2026-04-07

**Authors:** Electra Chatzidimitriou, Charis Styliadis, Katherine P. Rankin, Despina Moraitou, Panagiotis Ioannidis, Panagiotis D. Bamidis

**Affiliations:** 1 Laboratory of Medical Physics and Digital Innovation, Faculty of Health Sciences, School of Medicine, Aristotle University of Thessaloniki (AUTh), Thessaloniki, Greece; 2 Department of Cognition, Brain and Behavior, Faculty of Philosophy, School of Psychology, Aristotle University of Thessaloniki (AUTh), Thessaloniki, Greece; 3 2nd Department of Neurology, AHEPA University Hospital, Aristotle University of Thessaloniki (AUTh), Thessaloniki, Greece; 4 Department of Neurology, The Edward and Pearl Fein Memory and Aging Center, University of California, San Francisco, San Francisco, CA, United States

**Keywords:** artificial intelligence, dementia, functional neuroimaging, machine learning, MCI, MEG, neurophysiological biomarkers, risk prediction

## Abstract

**Introduction:**

Dementia imposes a substantial global healthcare burden, with rising prevalence and limited disease-modifying treatments. Early identification of at-risk individuals is critical for timely intervention and care planning. Magnetoencephalography (MEG) provides high-temporal-resolution measurements of neuronal activity, capturing subtle functional alterations that precede clinical symptoms. Artificial intelligence (AI), particularly machine learning (ML), can leverage MEG's rich spatiotemporal information to enhance diagnostic accuracy and dementia risk prediction. This scoping review synthesizes current evidence on AI-driven MEG analysis for the classification, prediction, and prognosis of MCI and dementia, focusing on methodological approaches, predictive performance, and translational potential.

**Methods:**

A systematic PubMed-MEDLINE search identified studies published between January 2015 and October 2025, capturing the last decade's rapid evolution of AI methodologies and their integration with neurophysiological research. Search terms combined MEG, AI, and ML with cognitive impairment and dementia. Eligible studies were peer-reviewed original research, involved human participants, employed MEG, and applied AI algorithms for classification or prediction. Extracted data included study population characteristics, MEG features, ML models, predictive biomarkers, and performance metrics.

**Results:**

Fourteen studies met eligibility criteria, covering populations from healthy controls to individuals with subjective cognitive decline, MCI, AD, and other dementias. MEG systems varied, with most studies employing 306-channel whole-head systems. ML algorithms ranged from traditional approaches, such as support vector machines and random forests, to deep learning architectures, including convolutional neural networks. Reported classification accuracies ranged from moderate (~60%) to high, with several studies achieving over 80% in distinguishing diagnostic categories or predicting MCI-to-AD progression. Key biomarkers included alterations in frequency-specific oscillatory activity, functional connectivity patterns, and large-scale network dynamics. Multimodal approaches integrating MEG with structural neuroimaging further improved predictive performance.

**Discussion/conclusions:**

Despite heterogeneity across study designs, AI-driven MEG analyses hold significant translational potential for early, non-invasive dementia prediction, enhancing diagnostic and prognostic accuracy. Advancing clinical translation will require standardized preprocessing pipelines, larger multicenter cohorts, and explainable AI frameworks. Future research should leverage next-generation MEG technologies, such as optically pumped magnetometers, to capture brain dynamics in ecologically valid, real-world scenarios. Integrating these data with AI-driven multimodal biomarkers will improve individualized risk prediction, early diagnosis, and therapeutic decision-making in dementia.

## Introduction

### Global dementia burden and the need for early detection

Dementia is a leading cause of disability and dependency among older adults, affecting more than 55 million people worldwide, a number projected to nearly triple by 2050 (Prince et al., 2014, 2015; Livingston et al., 2020, 2024). Beyond its profound personal and social impact, dementia imposes substantial economic and healthcare costs, driven by long-term care requirements, and caregiver burden (Wimo et al., 2016). Despite notable advances in understanding its neurobiological mechanisms, effective disease-modifying therapies remain limited. Consequently, identifying individuals at risk, before the onset of irreversible neurodegeneration, has become a major public health and research priority. Detecting neuronal dysfunction and cognitive decline at their earliest stages is therefore essential for enabling timely interventions, optimizing clinical management, and improving long-term outcomes (Prince et al., 2014, 2015; Livingston et al., 2020, 2024).

### Significance of biomarkers in dementia risk prediction

Given the critical importance of early identification, tools capable of detecting subtle neuronal changes are essential. Biomarkers—measurable indicators of underlying neuropathology—lie at the core of precision neurology (Ahmed et al., 2014), offering a powerful means to identify individuals at elevated risk of dementia. The preclinical and prodromal stages of dementia, during which neuronal dysfunction is detectable, but neurodegeneration remains minimal, represent a critical therapeutic window (Dubois et al., 2016). By capturing these early pathophysiological alterations, biomarkers can guide timely interventions, enable stratification for clinical trials, and support ongoing monitoring of disease progression (Ahmed et al., 2014).

Accurate risk stratification is particularly valuable for individuals with mild cognitive impairment (MCI), given the considerable heterogeneity in clinical trajectories within this population (Petersen et al., 2014; Ansart et al., 2020). Actionable biomarker information enables patients and clinicians to engage in proactive care planning and, at the population level, facilitates the efficient allocation of healthcare resources while informing the development of evidence-based health policies (Ramsey and Arnold, 2022; Wimo et al., 2016). Establishing reliable, accessible biomarkers capable of predicting cognitive decline and progression to dementia is therefore both a scientific and public health priority.

### Strengths of existing and emerging biomarker modalities

Substantial progress has been achieved in developing biomarkers that capture the molecular and structural hallmarks of dementia (Jack et al., 2018). Cerebrospinal fluid (CSF) analysis and positron emission tomography (PET) allow measurement of amyloid-beta deposition and tau pathology, often decades before symptom onset (Zetterberg, 2015; Simonsen et al., 2016; Cho et al., 2016). Fluorodeoxyglucose PET (FDG-PET) enables in vivo assessment of regional cerebral glucose metabolism, providing a sensitive measure of neuronal activity and functional integrity across brain regions (Mosconi, 2005; Kato et al., 2016). Single-photon emission computed tomography (SPECT) allows in vivo measurement of regional cerebral blood flow (rCBF) using radiotracers to evaluate perfusion patterns in the brain (Imokawa et al., 2024). Structural magnetic resonance imaging (MRI) offers high-resolution mapping of brain atrophy, supporting differential diagnosis and disease staging (Frisoni et al., 2010). Functional magnetic resonance imaging (fMRI) measures blood-oxygen-level-dependent (BOLD) signals to map changes in brain activity and functional connectivity (Logothetis, 2008). Electroencephalography (EEG) provides millisecond temporal resolution of neural oscillations, enabling the study of network-level changes in a non-invasive and cost-effective manner (Babiloni et al., 2011).

In parallel, blood-based biomarkers have emerged as a promising frontier for accessible and scalable dementia diagnostics (Hampel et al., 2018; Zetterberg, 2018; Chong et al., 2021; Grande et al., 2025). Notable examples include glial fibrillary acidic protein (GFAP), a marker of reactive astrogliosis associated with amyloid plaque accumulation; neurofilament light chain (NfL), which reflects axonal injury and neuronal damage characteristic of neurodegeneration; and plasma phosphorylated tau species (p tau181, p tau217), indicative of tau pathology and neurofibrillary tangle formation. Their minimal invasiveness and scalability make these biomarkers particularly attractive for population-level screening, longitudinal monitoring, and stratification in clinical trials (Hampel et al., 2018; Zetterberg, 2018; Chong et al., 2021; Grande et al., 2025).

Collectively, these modalities have brought dementia diagnostics closer to a biologically grounded framework for earlier detection, individualized risk assessment, and personalized care planning.

### Limitations of current biomarkers

Despite these advances, each biomarker modality faces distinct practical or methodological constraints that limit its suitability. CSF sampling is invasive and unsuitable for routine screening (Zetterberg, 2015; Simonsen et al., 2016); PET imaging is expensive, involves exposure to ionizing radiation, and is limited in accessibility (Cho et al., 2016; Mosconi, 2005); SPECT, although more widely available than PET, similarly requires radiotracer injection and associated radiation exposure, and offers lower spatial resolution; MRI primarily detects structural atrophy that typically emerges later in the disease course (Frisoni et al., 2010); fMRI, while offering valuable insights into functional connectivity, is costly, highly sensitive to motion and physiological noise, prone to interpretational challenges, and exhibits limited reproducibility across scanners and analytic pipelines, challenging its clinical applicability (Logothetis, 2008). EEG, while temporally precise, offers limited spatial specificity and is susceptible to artifacts (Horvath et al., 2017). Blood-based biomarkers, though promising, remain under investigation for diagnostic utility and still require further standardization and large-scale validation (Zetterberg, 2018).

These constraints have driven the search for non-invasive neurofunctional biomarkers capable of detecting subtle neuronal dysfunction at preclinical stages and suitable for routine, large-scale implementation in both research and clinical settings.

### Magnetoencephalography (MEG) as a functional neuroimaging biomarker

Magnetoencephalography (MEG) measures the minute magnetic fields generated by synchronous neuronal activity, offering millisecond temporal precision with improved spatial resolution over EEG (Hämäläinen et al., 1993; Baillet, 2017; Mandal et al., 2018). MEG directly captures neuronal oscillations across delta, theta, alpha, beta, and gamma frequency bands, providing a detailed characterization of cortical dynamics and functional connectivity (Mandal et al., 2018).

Accumulating evidence indicates that synaptic and network dysfunction precede measurable structural degeneration, making MEG a particularly valuable modality for detecting early signs of underlying neurodegenerative processes (Mandal et al., 2018). Early oscillatory disturbances, such as alpha-band slowing, reduced phase synchrony, and altered cross-frequency coupling, have been consistently linked to MCI and progression to dementia, reflecting disrupted communication within large-scale cortical networks that support cognitive abilities, such as memory, attention, and executive control (López et al., 2017; Mandal et al., 2018). Notably, these abnormalities often emerge before amyloid or tau deposition becomes measurable, positioning MEG as a sensitive tool for identifying preclinical and prodromal neural signatures of dementia (Mandal et al., 2018).

Compared with other functional neuroimaging modalities, MEG is silent, non-invasive, and well-tolerated, allowing repeated and longitudinal recordings, even in elderly or cognitively impaired individuals (Baillet, 2017; Mandal et al., 2018). Moreover, the growing availability of open-source MEG datasets, such as BioFIND, OMEGA, and Cam-CAN, have enhanced methodological consistency and reproducibility (Niso et al., 2015; Taylor et al., 2017; Vaghari et al., 2022a). Recent advances in optically pumped magnetometers (OPMs) have further expanded MEG's translational reach, enabling wearable, motion-tolerant MEG systems capable of recording brain activity in more naturalistic and clinical environments (Boto et al., 2018). These developments promise to transform MEG from a laboratory-based modality into a portable platform for precision monitoring of brain function in dementia.

### Artificial intelligence and machine learning in neuroimaging

Artificial intelligence (AI) and machine learning (ML) have revolutionized neuroimaging research by enabling automated, data-driven analysis of complex, high-dimensional brain data in health and pathology. Classical ML methods, including support vector machines (SVM) and random forests (RF), alongside more advanced deep learning architectures, such as convolutional neural networks (CNN) and graph neural networks (GNN), have achieved strong performance in diagnostic classification and disease-progression modeling (Grueso and Viejo-Sobera, 2021). These algorithms excel at uncovering non-linear relationships among imaging, cognitive and clinical features that traditional statistical methods often overlook. Furthermore, advances in explainable AI (XAI) methods, such as SHAP (Shapley Additive Explanations) and LIME (Local Interpretable Model-agnostic Explanations), have enhanced interpretability, transparency, and trust in AI-derived predictions (Tjoa and Guan, 2020).

### Integration of MEG and AI

The integration of MEG with AI methodologies unites the former's direct measurement of neuronal dynamics with the latter's analytical capacity to identify latent spatiotemporal patterns. This synergy enables the discovery of disease-related oscillatory and connectivity signatures that precede structural degeneration, thereby enhancing sensitivity for early diagnosis and individualized risk prediction (Ansart et al., 2020). ML models trained on MEG data have already demonstrated promising results in differentiating normal controls, individuals with MCI, and patients with different types of dementia (Pusil et al., 2019; Scheijbeler et al., 2022; Xu et al., 2021).

Recent translational frameworks, such as the “AI-Mind” (Haraldsen et al., 2024), emphasize the shift from descriptive to predictive neurophysiology, linking MEG's dynamic measures of network instability to computational models of synaptic efficiency and circuit communication. Moreover, combined with complementary modalities such as MRI, or blood-based biomarkers, as well as clinical and cognitive profiles, AI-driven MEG analytics can enable multimodal data fusion for integrative prediction and monitoring of disease progression. Together, these developments position AI-enhanced MEG as a cornerstone for next-generation non-invasive biomarkers that can transform dementia research and clinical care.

### Objectives and scope of the review

This scoping review synthesizes and critically evaluates current evidence on AI-driven MEG biomarkers for cognitive impairment and dementia risk prediction. It examines methodological approaches, predictive performance, and translational implications across recent studies, while also identifying key limitations that constrain reproducibility and clinical application, and outlining directions for future research. To our knowledge, this is the first review dedicated exclusively to the intersection of AI methodologies and MEG in the context of dementia, providing an integrated perspective that bridges computational neuroscience, biomarker discovery, and clinical translation.

## Methods

### Search strategy

An online search of the published literature was conducted to identify studies applying AI or ML techniques to MEG data in the context of cognitive decline, MCI, or dementia. The search covered the period from January 1, 2015, to October 17, 2025, to reflect the rapid evolution of AI methodologies and their integration with neurophysiological research over the past decade.

The bibliographic search was carried out in the PubMed (MEDLINE) database using the following query, which was applied to the title, abstract, or keyword fields:

(“magnetoencephalography” OR “MEG”) AND (“artificial intelligence” OR “machine learning” OR “deep learning” OR “neural network” OR “support vector machine” OR “random forest” OR “convolutional neural network”) AND (“dementia” OR “Alzheimer's disease” OR “mild cognitive impairment” OR “AD” OR “MCI” OR “cognitive decline”).

### Eligibility criteria

Studies were eligible for inclusion if they:

(1) involved human participants with cognitive impairment or dementia, including individuals with subjective cognitive decline (SCD), MCI, or any type of dementia;
(2) used MEG as the primary neuroimaging modality; and
(3) applied AI or ML algorithms to MEG data for the purposes of classification, prediction, or prognosis of cognitive impairment or dementia.

Only peer-reviewed original research articles published in English were included to ensure scientific quality and facilitate accurate data extraction. Eligible studies were required to report outcomes related to model performance metrics, predictive features, or clinically relevant biomarkers.

Exclusion criteria included:

- studies not employing MEG or AI techniques
- studies involving animals or computational simulations
- non-original research articles such as conference abstracts without full text availability, reviews, commentaries, or opinion papers, and
- studies published in languages other than English.

### Critical appraisal and synthesis approach

Given the substantial heterogeneity across study designs, analytical frameworks, and reported outcomes, an integrative synthesis approach was adopted to enable a critical appraisal and systematic evaluation of the included studies. This method facilitated a qualitative assessment of the existing evidence, emphasizing conceptual patterns, methodological trends, and emerging insights rather than quantitative aggregation.

Key studies were analyzed with respect to their objectives, participant characteristics, MEG acquisition and preprocessing methods, ML algorithms, validation strategies, and main findings, with particular emphasis placed on the predictive features or biomarkers identified and the reported performance metrics (e.g., accuracy, area under the curve [AUC], sensitivity, specificity, and F1 score).

Through this approach, this review sought to provide a comprehensive overview of the current state of research, highlighting the potential of MEG-derived features combined with ML techniques for early dementia risk prediction and classification, while identifying key methodological approaches, recurring challenges, and directions for future investigation in the field.

## Results

The literature search in the PubMed (MEDLINE) database identified a total of 29 potentially relevant studies (Maestú et al., 2015; Amezquita-Sanchez et al., 2016; Azami et al., 2016; Rawool, 2016; Ranasinghe et al., 2017; James et al., 2018; Sitnikova et al., 2018; Koelewijn et al., 2019; Pusil et al., 2019; Yang et al., 2019; Ansart et al., 2020; Ikeda et al., 2020; Lopez-Martin et al., 2020; Cope et al., 2021; Grueso and Viejo-Sobera, 2021; Shaw et al., 2021; Xifra-Porxas et al., 2021; Xu et al., 2021; Yang et al., 2021; Scheijbeler et al., 2022; Vaghari et al., 2022a,b; James et al., 2023; Revilla-Vallejo et al., 2023; Luppi et al., 2024; Pascarella et al., 2024; Farag et al., 2025; Javed et al., 2025; Ramirez et al., 2025).

Following the application of predefined eligibility criteria, several studies were excluded. More specifically, seven studies were review articles that did not present original data or empirical analyses and were therefore excluded, as this scoping review focused only on original research (Yang et al., 2019; Ansart et al., 2020; Cope et al., 2021; Grueso and Viejo-Sobera, 2021; Rawool, 2016; Pascarella et al., 2024; Farag et al., 2025). An additional three studies were excluded, because they did not incorporate ML methodologies, which represented a core inclusion criterion (Ranasinghe et al., 2017; James et al., 2023; Revilla-Vallejo et al., 2023). One study was removed as it was a conference proceeding (Azami et al., 2016), another because it did not employ MEG data (Luppi et al., 2024), and three involved only healthy participants without cognitive impairment or dementia (James et al., 2018; Xifra-Porxas et al., 2021; Ramirez et al., 2025), placing them outside the scope of this review.

In total, 14 studies met all predefined eligibility criteria (Maestú et al., 2015; Amezquita-Sanchez et al., 2016; Sitnikova et al., 2018; Koelewijn et al., 2019; Pusil et al., 2019; Ikeda et al., 2020; Lopez-Martin et al., 2020; Shaw et al., 2021; Xu et al., 2021; Yang et al., 2021; Scheijbeler et al., 2022; Vaghari et al., 2022a,b; Javed et al., 2025). An overview of the key characteristics of the included studies is presented in Table 1, which summarizes details on study populations, MEG acquisition parameters, extracted features, ML approaches, prediction targets, and principal findings.

**Table 1:** Overview of eligible MEG-based studies applying AI approaches for the prediction and classification of cognitive impairment and dementia: modalities, extracted features, and key outcomes.

References	Sample/ population	MEG system/ modality	MEG features extracted	AI/ML method	Prediction target/ outcome	Key findings
Scheijbeler et al. (2022)	*n* = 392: diagnosed with Subjective Cognitive Decline (SCD) = 105, Mild Cognitive Impairment (MCI) = 45, Alzheimer's Disease (AD) = 127, Dementia with Lewy Bodies (DLB) = 27, Frontotemporal Dementia (FTD) = 33, and Psychiatric Illness (Psy) = 55	306-channel whole-head Vectorview MEG system (Elekta Neuromag Oy, Helsinki, Finland; source localized resting state; eyes closed)	• Relative power in canonical frequency bands (delta 0.5–4 Hz, theta 4–8 Hz, alpha-1 8–10 Hz, alpha-2 10–13 Hz, beta 13–30 Hz, gamma 30–48 Hz) • Functional connectivity via corrected Amplitude Envelope Correlation (AEC-c) between regions of interest (ROIs), with pairwise orthogonalization to correct for spatial leakage • Source-localized to 90 ROIs using beamforming; features computed per ROI, aggregated across 12 subregions (left/right frontal, central, temporal, parietal, occipital, subcortical) and globally, resulting in 156 features per subject • Features averaged across 10 artifact-free, eyes-closed epochs	Random Forest (RF) classifier with probability-based diagnostic profiles (ensemble of 500 bootstrap-aggregated decision trees; feature selection and feature importance assessed via Gini impurity index; trained on 90% of data with random under-sampling to handle class imbalance; class probabilities estimated via tree votes; final diagnosis by majority vote; tested on 10% unseen data; performance evaluated for 6-class multi-dementia and lower-order classification tasks)	Multi-class classification of six diagnostic groups: SCD, MCI, AD, DLB, FTD, and Psy	• Overall classification: MEG-based classification achieved a balanced accuracy of 41% and a multi-class area under the curve (AUC) of 0.75 for 6-class diagnosis • Feature importance: classification performance relied primarily on posterior relative delta, theta, and beta power, and amplitude-based functional connectivity in beta and gamma frequency bands • Best performance: DLB—sensitivity 100%, precision 20% and AD: sensitivity 51%, precision 90% • Worst performance: FTD—sensitivity 11%, precision 3% • Diagnostic profiles: probability estimates for each of the six diagnostic categories were generated for each participant, facilitating clinical interpretation • Clinical relevance: MEG biomarkers can provide a non-invasive tool for early and accurate differentiation of dementia subtypes, potentially supporting diagnosis in memory clinics and informing timely treatment planning
Yang et al. (2021)	*n* = 40: 20 with MCI, 20 healthy controls (HC)	306-channel Vectorview MEG system (Elekta Neuromag; resting state; eyes closed)	• Wavelet-based source-level features computed from MEG signals using Wavelet Packet Decomposition (WPD), covering delta, theta, alpha, beta, and gamma frequency bands (0.5–80 Hz) per ROI • Functional connectivity quantified via Magnitude Squared Coherence (MS-COH) between 10 selected ROIs, (frontal, temporal, parietal, occipital, central, left/right hemispheres), producing pairwise connectivity values • Source-localized to 10 ROIs (hyper- and hypo-connected groups), with feature vectors aggregated across participants for classification	Support Vector Machine (SVM), Linear Discriminant Analysis (LDA) and k-Nearest Neighbors (k-NN) classifiers	Classify MCI vs. HC using MEG-based wavelet functional connectivity features	• Hyper-connected ROIs outperformed hypo-connected ROIs for MCI detection, highlighting the importance of identifying overactive brain regions in MCI • Best average recognition rate: 93.83% achieved using source-reconstructed signals from hyper-connected ROIs, indicating excellent detection performance • Leave-One-Out Cross-Validation (LOOCV) Accuracy: 83.80%, showing robust classification performance even with small sample sizes • Multi-resolution wavelet biomarkers: demonstrated significant potential for MCI detection, suggesting that time-frequency features could enhance classification accuracy • Wavelet neuromarkers outperform conventional features: the proposed multi-resolution wavelet features improved classification performance by ~15% compared to standard wavelet-based approaches • Research vs. clinical evaluation gap: results highlight a substantial performance drop when moving from research-friendly methods to clinically valid evaluation, underscoring the need for clinically grounded validation
Amezquita-Sanchez et al. (2016)	*n* = 37: 18 with MCI, 19 HC	148-channel whole-head magnetometer (MAGNES 2,500 WH, 4D Neuroimaging, San Diego, CA; working memory task)	• Complete Ensemble Empirical Mode Decomposition (CEEMD) into adaptive sub-bands • Permutation Entropy (PE) • Feature selection via analysis of variation (ANOVA)	Enhanced Probabilistic Neural Network (EPNN)	Classify MCI vs. HC	• The proposed methodology (CEEMD + PE + EPNN) successfully distinguished MCI from HC using MEG data during a working memory task with a high accuracy of 98.4% • First application of CEEMD for detecting impaired working memory in MCI using MEG
Xu et al. (2021)	*n* = 129: 53 neurotypical controls (NC), 48 stable MCI (sMCI), 28 progressive MCI (pMCI); age range: 65–80 years old	306-channel Elekta Vectorview MEG system (204 planar gradiometers, 102 magnetometers; resting-state, eyes closed, 3–5 min recordings; source reconstructed to 68 ROIs from Desikan-Killiany atlas)	• Functional connectivity via normalized mutual information (MI) in alpha band (8–12 Hz) between all 68 ROIs • Constructed subject-specific undirected weighted MEG brain networks (68 × 68 matrices) • Multiple Graph Gaussian Embedding (MG2G) generated node-wise multivariate Gaussian embeddings (mean + variance) • Low-dimensional latent features (embedding size L = 16) representing network structure per node	Traditional ML classifiers applied to MG2G-generated embeddings, including k-NN, Linear SVM, RBF (Radial Basis Function kernel) SVM, Gaussian Process, Decision Tree, RF, Neural Net, AdaBoost, Naive Bayes, and Quadratic Discriminant Analysis (QDA; 5-fold CV, applied to 3-class and 2-class classification tasks)	Predict progression from MCI to AD and compare stable vs. progressive MCI and NC vs. MCI stages	• MG2G embeddings captured highly informative MEG network features and consistently outperformed the node2vec baseline • Classification performance: NC/sMCI/pMCI = 61%, NC/sMCI = 79%, sMCI/pMCI = 78%, NC/pMCI = 82% • Significant NC vs. sMCI differences detected in 34 brain regions, predominantly in frontal and temporal lobes • Significant sMCI vs. pMCI differences in 18 regions, mainly in frontal, temporal, and parietal lobes • Significant NC vs. pMCI differences in 19 regions, largely in frontal, temporal, and parietal lobes • Hemispheric asymmetry observed, with more regions affected in the left hemisphere • MG2G captures latent hierarchical and non-linear MEG network patterns, enabling effective early AD progression prediction even with moderate sample sizes
Pusil et al. (2019)	*n* = 54: 27 sMCI, 27 pMCI; Age range: 65–80 years old; Longitudinal study with follow-up of approximately 3 years	306-channel Elekta Neuromag Vectorview MEG system (resting-state, eyes closed; 5 min; source reconstructed to ROI time series)	• Source-reconstructed time series from 90 ROIs • Dynamic functional connectivity across δ, θ, α, β, γ bands via Phase Coupling Estimation (PCE) • Temporal decoupling via Dynamic Time Warping (DTW) between first and second MEG sessions • ROI-level representative time series extracted via CENT and Principal Component Analysis (PCA)	Multi-cluster feature selection (MCFS) with SVM using RBF kernel; 5-fold CV; multiplex multi-frequency integration	Predict conversion from MCI to AD (sMCI vs. pMCI)	• Multi-frequency dynamic functional connectivity features achieved 100% classification accuracy for sMCI vs. pMCI (CENT-based features) • Most discriminative connections involved Default Mode Network (DMN), cingulo-opercular (CO), fronto-parietal (FP), sensorimotor, thalamo-related, and olfactory networks • Gamma (γ) band alone achieved high classification accuracy (94%) • PCE outperformed conventional bivariate phase-based connectivity estimators • Dynamic connectivity features were more informative than static measures • Higher DTW values indicated greater longitudinal disruption of functional connectivity (temporal decoupling) in pMCI, whereas lower DTW values reflected more stable and temporally coupled connectivity, potentially suggesting compensatory network dynamics for cognitive loss • Strong correlations observed between DTW-derived features and changes in global cognition (MMSE) • A multi-frequency dynamic network integration approach enabled the construction of a sensitive MEG-based connectome biomarker that accurately predicts conversion from MCI to AD
Koelewijn et al. (2019)	*n* = 183: healthy young adults genotyped for APOE-ε4 (51 APOE-ε4 carriers, 108 non-carriers, 24 participants excluded due to unsuccessful genotyping); a separate cohort included AD patients (*n* = 14) and age-matched elderly controls (*n* = 11)	275-channel CTF radial gradiometer MEG system (resting-state; eyes open/closed paradigm; 5 min whole-head recordings; 29 reference channels; sampled at 1,200 Hz; synthetic third-order gradiometers)	• Source-localized oscillatory amplitude connectivity in multiple frequency bands (delta, theta, alpha, beta, and gamma) using amplitude envelope correlations • Combined vector-sum connectivity across frequencies	SVM classifier with LOOCV; features: top 20% of valid connectivity edges across frequency bands; permutation testing for significance	• Classify APOE-ε4 carriers vs. non-carriers • Classify AD patients vs. elderly controls • Cross-prediction: test whether classifier trained on healthy young APOE-ϵ4 carriers generalizes to AD patients based on MEG connectivity matrices	• Young APOE-ε4 carriers showed hyperconnectivity predominantly in alpha/beta bands, with a right-hemisphere bias involving parietal and precuneus regions overlapping with the DMN, and gamma (40–160 Hz) hyperactivity in right-lateralized frontal, temporal and parietal regions • AD patients exhibited hypoconnectivity predominantly in parieto-temporal areas compared to age-matched elderly controls, particularly in alpha and beta bands, within a widespread bilateral network that partially overlapped with regions showing hyperconnectivity in young APOE-ε4 carriers • The SVM classifier achieved 63.5% AUC for APOE-ϵ4 carriers vs. non-carriers and 77.6% AUC for AD vs. elderly controls • Cross-prediction analysis demonstrated partial regional overlap between young APOE-ε4 hyperconnectivity patterns and AD-related hypoconnectivity • Findings support a model in which early-life hyperconnectivity may precede later large-scale network disconnection in AD • Results indicate that MEG-derived connectivity patterns may provide a potential early biomarker of AD-related pathophysiology
Vaghari et al. (2022a)	*n* = 324: 158 individuals with MCI and 166 HC; multi-site dataset (the “BioFIND” project)	306-channel Elekta Neuromag Vectorview MEG system (resting-state; eyes closed; 2–13 min recordings; 102 magnetometers and 204 planar gradiometers; multi-site acquisition; structural T1-weighted MRI acquired for most participants for source reconstruction; data stored in BIDS format)	• Sensor-level spectral power (normalized Alpha 8–12 Hz and Beta 13–30 Hz across 306 channels) • Source-level spectral power via beamforming to 38 cortical ROIs • Functional connectivity via AEC between 38 ROI time-series (orthogonalised) • Covariate-adjusted features (e.g., site, age, sex, education, head motion) assessed	SVM classifier; 10-fold CV; averaged over 100 permutations; performance evaluated via balanced accuracy; analyses benchmark different feature types and confound adjustments	• Classify MCI vs. HC • Compare classification performance across feature types (sensor power, source power, connectivity) • Assess the impact of covariates and confounds on classification	• MEG detected early changes in brain activity potentially related to neurodegeneration, even before structural MRI changes were evident • Classification accuracy for distinguishing MCI from HC ranged from 60% to 68%, depending on covariate inclusion • Using all MEG sensors without covariates, 67.8% accuracy was achieved • Accuracy decreased to 66.9% when adjusting for experimental/technical confounds (e.g., recording site, head motion), and further to 59.4% after adjusting for demographic covariates (age, sex, education) • Confounds such as site, age, and education significantly influenced classification performance • Sensor-level power features (alpha and beta band) outperformed source power and source connectivity features for classification, achieving 66.1% accuracy, compared with 65.2% and 64.8%, respectively • This study demonstrated the feasibility of classifying MCI vs. HC using MEG data from a large multi-site dataset • BioFIND provides the largest publicly available resting-state MEG dataset for dementia research, offering a valuable resource to investigate the neurophysiological underpinnings of dementia-related pathophysiology • Results support the utility of MEG in detecting early functional brain changes associated with MCI
Vaghari et al. (2022b)	*n* = 307: 144 MCI cases and 163 elderly HC; multi-site dataset (the “BioFIND” project)	306-channel Elekta Neuromag Vectorview MEG system (resting-state; eyes closed; ≥120 s used; down-sampled to 500 Hz; band-pass filtered 0.5–98 Hz; 102 magnetometers and 204 planar gradiometers; sensor-level analysis; multi-site acquisition; structural T1-weighted MRI included for multimodal analysis)	• Sensor-level variance and covariance features from magnetometers and planar gradiometers • Features across six frequency bands: delta (2–4 Hz), theta (4–8 Hz), alpha (8–12 Hz), beta (12–30 Hz), low gamma (30–48 Hz), high gamma (52–86 Hz) • MRI features: gray-matter volume across 110 ROIs (Harvard–Oxford Atlas) • Confound kernels: site, age, sex, education, time-of-day, head motion, distance from sensors	SVM with Multiple Kernel Learning (MKL); nested CV (5-fold, repeated 1,000 times); comparison of “Early,” “Intermediate,” and “Late” combination strategies of MRI and MEG modalities	• Classify MCI vs. elderly HC • Evaluate whether MEG adds complementary information beyond MRI • Compare multimodal integration strategies	• Combining MEG and MRI data improved classification performance compared to either modality alone • MEG alone achieved 68% accuracy, while MRI alone achieved 71% • Late fusion of MEG and MRI kernels achieved the highest classification accuracy (77%) outperforming intermediate and early approaches • MEG improved MRI classification in 97.5% of permutations • Gamma frequency bands provided the most discriminative features • Covariance features (especially planar gradiometers) outperformed variance features • Frequency band selection was more impactful than MEG sensor type • Confounds alone achieved 64.5% accuracy, indicating strong influence • Kernel-based multimodal approach effectively handled confounds • The study confirmed the complementary value of MEG in enhancing MCI detection beyond MRI
Ikeda et al. (2020)	*n* = 47: 20 early-stage AD patients (6 with MCI due to AD, 14 with probable AD dementia) and 27 HC	160-channel whole-head MEG system (PQA160C, Yokogawa Electric Corporation; coaxial gradiometers; resting-state; eyes closed and eyes open conditions; 2 min recordings per condition; sampling rate 1,000 Hz; bandpass filter 0.25–500 Hz; MRI co-registration; source reconstruction using Tikhonov-regularized minimum-norm estimation)	• Source-level spectral power across 68 cortical regions (Desikan–Killiany atlas) • Absolute power during eyes closed (EC) and eyes open (EO) conditions • Whole cerebral normalization (WCN) power in 5 frequency bands (θ1: 4–6 Hz, θ2: 6–8 Hz, α1: 8–10 Hz, α2: 10–13 Hz, β: 13–20 Hz) during EC and EO conditions • Difference of the absolute powers between the EC and EO conditions (the EC-EO difference) • WCN value of the EC–EO difference	SVM classifier; polynomial kernel; 6-fold CV; receiver operating characteristic curve (ROC) analysis	• Classify early-stage AD vs. HC • Evaluate discriminative value of features from EC, EO, and EC–EO conditions	• Significant differences in WCN power values were observed between AD and HC, particularly during EO condition and EC–EO differences • The model achieved classification accuracy of 83% (Sensitivity: 70%, Specificity: 93%, with an AUC of 0.91) • Increased θ and α band activity in temporo-parietal regions and reduced α in frontal regions in AD • Altered EC–EO modulation indicates disrupted network reactivity in AD • MEG source-level features can effectively discriminate early-stage AD from HC • MEG is a promising biomarker for detecting cortical activity alterations in early-stage AD
Sitnikova et al. (2018)	*n* = 20: 10 patients with AD and 10 HC	306-channel Elekta Neuromag VectorView MEG system (102 magnetometers and 204 planar gradiometers; resting-state fixation; 3 consecutive 3 min recordings per participant, ~9 min total; 0.03–330 Hz; 1,000 Hz sampling; source reconstruction using a linearly constrained minimum variance-LCMV scalar beamformer)	• Temporal dynamics of spontaneous neural synchrony, focusing on oscillatory (4–30 Hz) and ultra-slow (< 0.1 Hz) activity patterns in the DMN and other large-scale networks, such as the dorsal attention network (DAN), the visual network (VisN), the sensorimotor network (SMN), and the left associative network (LAN) • Symmetric orthogonalization to reduce signal leakage • Temporal metrics: fractional occupancy, fractional count, mean lifetime, transition probabilities	Hidden Markov Model (HMM) for unsupervised detection of transient network states	Identify and quantify short timescale abnormalities in functional neural network synchrony in AD vs. HC	• Short timescale abnormalities in spontaneous neural synchrony were identified in AD, particularly within the DMN • Individuals with AD showed shorter DMN and DAN state durations and less robust oscillatory amplitudes in posterior DMN hubs • Abnormal state transitions between HMM-derived brain states: increased DMN → LAN, decreased VisN → DMN • Fast-timescale network disruptions persisted despite ultra-slow modulation • Altered temporal patterns of brain-state visits suggest impaired coordination and stability of large-scale functional networks in AD • Findings support the hypothesis that dendritic depolarizations and modulations of cortical excitability may contribute to disrupted neural synchrony in AD • Source-level MEG metrics may serve as sensitive indicators of network synchrony deficits in AD
Maestú et al. (2015)	*n* = 184 total: 102 individuals with MCI and 82 HC; multicenter dataset from 5 MEG centers (Madrid, Cambridge, Helsinki, Obu-Nagoya, Pittsburgh); dataset structure: dataset 1 (78 MCI, 54 HC), Dataset 2 (13 MCI, 15 HC), Dataset 3 (11 MCI, 13 HC)	306-channel Elekta Neuromag VectorView MEG system (102 magnetometers and 204 planar gradiometers; analysis restricted to 102 magnetometers); resting-state eyes closed; 3–5 min recordings; sampling rate 1,000 Hz (1,001.6 Hz in Helsinki); bandpass 0.03–330 Hz; temporal signal space separation (tSSS) noise reduction (MaxFilter) was applied; epochs of 2 s; analysis performed in sensor space	• Functional connectivity between magnetometer pairs using MI • Connectivity matrices across frequency bands: theta (4–8 Hz), alpha (8–12 Hz), beta (12–30 Hz), gamma (30–45 Hz), broadband (2–45 Hz) • Connectivity computed for 5,151 sensor pairs • Summary statistics across epochs: median, maximum, minimum, interquartile range • 20,604 features per subject and frequency band	Clinical Data Partitioning (CliDaPa) machine-learning framework combining feature selection (χ^2^ filter) with multiple classifiers: RF, Bayesian Network, C4.5 decision tree, K-NN, Logistic Regression, and SVM. Bootstrap validation (0.632, 100 iterations) and external validation across datasets	• Classify MCI vs. HC using MEG functional connectivity patterns • Evaluate generalizability across multiple MEG centers	• MEG functional connectivity patterns successfully discriminated MCI from HC at the individual level • Internal validation accuracy 79% (sensitivity 83%, specificity 72%) • External validation across centers achieved 82% accuracy (sensitivity 92%, specificity 73%) • Broadband connectivity produced the most robust classification performance • MCI patients showed hypersynchronization in fronto-parietal and interhemispheric connections, potentially reflecting a compensatory neural response • The links between sensor pairs that best classified the groups were bilateral anterio-posterior and mid-anterior interhemispheric connections • The hypersynchronization pattern was consistent across the five MEG centers, demonstrating cross-site reliability • Findings support the view that early AD may represent a disconnection syndrome involving synaptic dysfunction • MEG functional connectivity may serve as a useful non-invasive biomarker of preclinical synaptic disruption
Shaw et al. (2021)	*n* = 73: 33 with behavioral variant frontotemporal dementia (bvFTD), 40 age- and sex-matched HC	306-channel Elekta Neuromag VectorView MEG system (102 magnetometers and 204 planar gradiometers); 500 Hz sampling; passive auditory oddball paradigm; epochs −100–300 ms; bandpass 1–40 Hz; source localization to bilateral inferior frontal gyrus (IFG), superior temporal gyrus (STG), and primary auditory cortex (A1)	• Dynamic causal modeling (DCM) of canonical microcircuits (CMC) • Layer-specific and region-specific parameters • Intrinsic (within-region) and extrinsic (between-region) connectivity • Layer-by-node contributions (J) • Evoked response field (ERF) features: mismatch negativity (MMN) amplitude and latency (80–200 ms) at each source	SVM with permutation-based LOOCV; classifiers trained separately on: (1) effective connectivity strengths between nodes, (2) layer-by-node contributions (J), (3) MMN amplitudes	• Classify bvFTD vs. HC • Compare predictive value of DCM parameters vs. conventional ERF measures	• The optimal model included right IFG top-down input, with no left IFG or interhemispheric connections • Layer-by-node (J) parameters achieved excellent discrimination (99.6% classification accuracy) between bvFTD and HC (sensitivity = 99.2%, specificity = 100%), outperforming MMN amplitudes alone (~60% accuracy) • bvFTD patients showed reduced contributions from deep pyramidal cells (layers L5/6) in STG and altered superficial pyramidal cell (L2/3), consistent with histopathological laminar cell loss in bvFTD • Changes in intrinsic connectivity indicate impaired predictive coding of auditory events in bvFTD • Generative model parameters provided mechanistic insight into laminar- and region-specific degeneration beyond conventional ERF measures • Model-based MEG parameters provided a direct physiological index of cortical microcircuit degeneration in bvFTD • Findings support use of DCM-based MEG metrics as mechanistic biomarkers for experimental medicine studies and early-phase clinical trials in bvFTD
Lopez-Martin et al. (2020)	*n* = 132: 78 MCI, 54 HC	306-channel Elekta Neuromag MEG system; resting-state recording (eyes closed) for 4 min; sampling rate 1,000 Hz; tSSS noise reduction was applied; band-pass filtering 2–45 Hz; analysis focused on 102 magnetometers; signals segmented into 2-s epochs; artifact rejection using FieldTrip	• Functional connectivity features computed using MI between MEG sensor pairs; 25,755 features per subject • Connectivity strength statistics across epochs: mean, median, standard deviation, mean absolute deviation, range (maximum-minimum) • Feature selection methods: RF variable importance, Boruta, and Recursive Feature Elimination (RFE) • Selected connectivity features used as input for Convolutional Neural Network (CNN)-based classification	Randomized 2D-CNN ensemble with shared weights and random feature permutations; compared with 1D-CNN, 2D-CNN, multilayer perceptron, extreme learning machine, and classical ML models (RF, SVM, logistic regression, naïve Bayes, gradient boosting, Gaussian process classifier); nested CV	Classify MCI vs. HC	• The proposed randomized 2D-CNN ensemble achieved the best performance among the tested models and effectively handled high-dimensional and noisy MEG functional connectivity data with a limited sample size • The model achieved state-of-the-art performance, with an F1-score of 0.92 for classifying MCI vs. HC, outperforming conventional ML methods and other CNN architectures • Random permutations of connectivity features improved exploration of feature interactions and helped reduce overfitting in high-dimensional data • Findings demonstrated that MEG functional connectivity features combined with deep learning approaches can effectively identify MCI, supporting their potential use for early detection of prodromal AD
Javed et al. (2025)	• Cross-sectional: *n* = 343 (HC = 116, SCD = 85, MCI = 142) • Longitudinal: *n* = 45 MCI (stable MCI = 22; progressive MCI = 23)	306-channel Vectorview MEG system (102 magnetometers, 204 planar gradiometers; Elekta Neuromag Oy; source-reconstructed resting-state; eyes closed)	• Functional excitation-inhibition ratio (fE/I) • Long-Range Temporal Correlations (LRTCs) via Detrended Fluctuation Analysis (DFA) • fE/I and DFA features averaged across parcels and canonical frequency bands (theta 4–7 Hz, alpha 7–12 Hz, beta 12–30 Hz)	• kNN classifier: minimal-optimal features selected with minimum-Redundancy-Maximum-Relevance (mRMR), trained using LOOCV, hyperparameters optimized via grid search, tested on 25% unseen data; feature importance assessed using Shapley Additive Explanations (SHAP) plots • Validation with SVM using a Gaussian kernel	Classification of early AD stages: • HC vs. SCD • HC vs. MCI • SCD vs. MCI Prediction of disease progression from MCI to AD in the longitudinal cohort	• LRTCs exhibited localized attenuation within specific spectral bands and anatomical regions in SCD, with this disruption becoming more widespread in MCI; fE/I was elevated in the MCI but not in the SCD group, reflecting distinct functional alterations across disease stages • Machine learning analysis of functional MEG and structural MRI data showed that LRTCs and fE/I were the most informative predictors for classifying SCD, whereas MTL volumes best distinguished MCI • The model achieved strong classification performance, with mean accuracies of 81.67% for training and 75.67% for test sets across all binary classification tasks, and mean ROC AUC values of 84.67% and 77% for training and test sets, respectively • Model performance metrics demonstrated reliable discrimination of SCD from both HC and MCI, with precision ranging 61%−82% and recall 69%−86% across training and test sets, highlighting the ability of MEG-based measures to detect very early stages of the AD continuum
						• Both LRTCs and fE/I predicted disease progression over time, distinguishing stable from progressive MCI cases: longitudinal analysis revealed attenuated DFA exponents and increased fE/I values in the progressive MCI cases, accompanied by greater cognitive impairment, highlighting early MEG neurophysiological changes associated with progression to dementia • Alterations in LRTCs and fE/I reflect shifts toward supercritical brain dynamics, marking early AD processes

A1, primary auditory cortex; AEC-c, Amplitude Envelope Correlation (corrected); AD, Alzheimer's Disease; ANOVA, Analysis of Variation; AUC, Area Under the Curve; BIDS, Brain Imaging Data Structure format; bvFTD, Behavioral Variant Frontotemporal Dementia; CEEMD, Complete Ensemble Empirical Mode Decomposition; CENT, centroid; CliDaPa, Clinical Data Partitioning; CMC, Canonical Microcircuits; CNN, Convolutional Neural Network; CO, Cingulo-Opercular; CV, Cross-Validation; DAN, Dorsal Attention Network; DCM, Dynamic Causal Modeling; DFA, Detrended Fluctuation Analysis; DLB, Dementia with Lewy Bodies; DMN, Default Mode Network; DTW, Dynamic Time Warping; EC, Eyes Closed; EO, Eyes Open; EPNN, Enhanced Probabilistic Neural Network; ERF, Evoked Response Field; FP, Fronto-Parietal; FTD, Frontotemporal Dementia; fE/I, functional Excitation-Inhibition ratio; HC, Healthy Controls; IFG, Inferior Frontal Gyrus; HMM, Hidden Markov Model; kNN, k-Nearest Neighbors; LAN, Left Associative Network; LCMV, Linearly Constrained Minimum Variance scalar beamformer; LDA, Linear Discriminant Analysis; LOOCV, Leave-One-Out Cross-Validation; LRTCs, Long-Range Temporal Correlations; MEG, Magnetoencephalography; MCFS, Multi-Cluster Feature Selection; MCI, Mild Cognitive Impairment; MG2G, Multiple Graph Gaussian Embedding; MI, Mutual Information; MKL, Multiple Kernel Learning; ML, Machine Learning; MMN, Mismatch Negativity; MMSE, Mini-Mental State Examination; MRI, Magnetic Resonance Imaging; mRMR, minimum-Redundancy-Maximum-Relevance; MTL, Medial Temporal Lobe; MS-COH, Magnitude-Squared Coherence; NC, Neurotypical Controls; PCA, Principal Component Analysis; PCE, Phase Coupling Estimation; PE, Permutation Entropy; pMCI, progressive Mild Cognitive Impairment; Psy, Psychiatric Illness; QDA, Quadratic Discriminant Analysis; RBF, Radial Basis Function kernel; RF, Random Forest; RFE, Recursive Feature Elimination; ROC, Receiver Operating Characteristic Curve; SCD, Subjective Cognitive Decline; SHAP, Shapley additive explanations; sMCI, stable Mild Cognitive Impairment; SMN, Sensorimotor Network; STG, Superior Temporal Gyrus; SVM, Support Vector Machine; tSSS, temporal Signal Space Separation; VisN, Visual Network; WCN, Whole Cerebral Normalization; WPD, Wavelet Packet Decomposition.

### Characteristics of the included studies and study populations

The 14 included studies encompassed a wide range of participant populations, from healthy controls (HC) to individuals with SCD, MCI, AD, dementia with Lewy bodies (DLB), frontotemporal dementia (FTD), and psychiatric conditions (Maestú et al., 2015; Sitnikova et al., 2018; Shaw et al., 2021; Ikeda et al., 2020; Xu et al., 2021; Scheijbeler et al., 2022; Javed et al., 2025). Several studies also examined preclinical or risk populations, including young carriers of the APOE ε4 allele, a major genetic risk factor for AD (Koelewijn et al., 2019). Sample sizes varied substantially, ranging from small proof-of-concept cohorts of approximately 20 participants (Sitnikova et al., 2018; Ikeda et al., 2020) to large multi-site datasets of up to 392 individuals (Scheijbeler et al., 2022).

Across the included literature, most studies focused on diagnostic classification tasks, particularly distinguishing MCI or AD from cognitively HC (Amezquita-Sanchez et al., 2016; Maestú et al., 2015; Yang et al., 2021; Lopez-Martin et al., 2020; Vaghari et al., 2022a). A smaller subset examined disease progression or risk stratification, such as predicting conversion from stable MCI to progressive MCI or identifying early neurophysiological alterations in individuals at genetic risk (Pusil et al., 2019; Xu et al., 2021; Koelewijn et al., 2019; Javed et al., 2025). For example, longitudinal designs were employed to predict conversion from MCI to AD (Pusil et al., 2019; Xu et al., 2021), whereas other studies investigated early-stage neurophysiological changes across the preclinical spectrum from SCD to MCI (Javed et al., 2025).

Only a limited number of studies attempted multi-class diagnostic classification across several dementia subtypes. One large cohort study evaluated six diagnostic groups (SCD, MCI, AD, DLB, FTD, and psychiatric disorders), demonstrating the feasibility—but also the increased complexity—of multi-class classification tasks using MEG features (Scheijbeler et al., 2022). Overall, the reviewed literature demonstrates substantial heterogeneity in both study objectives and target populations, reflecting the diverse ways in which ML methods have been applied to MEG data in dementia research.

### MEG acquisition paradigms and systems

MEG recordings across studies were obtained using whole-head systems with sensor arrays ranging from 148 to 306 channels. The majority of studies used 306-channel systems, such as the Elekta Neuromag Vectorview, which provide high spatial and temporal resolution through combined magnetometer and planar gradiometer measurements (Maestú et al., 2015; Sitnikova et al., 2018; Lopez-Martin et al., 2020; Shaw et al., 2021; Xu et al., 2021; Yang et al., 2021; Scheijbeler et al., 2022; Vaghari et al., 2022a; Javed et al., 2025). Other systems included CTF radial gradiometer arrays (Koelewijn et al., 2019), Yokogawa coaxial gradiometer systems (Ikeda et al., 2020), and 148-channel magnetometer systems (Amezquita-Sanchez et al., 2016). Recording durations typically ranged between 3 and 10 min, with sampling rates commonly between 500 and 1,000 Hz.

A notable methodological pattern across the literature is the predominant use of resting-state paradigms. The majority of studies recorded spontaneous brain activity during eyes-closed or eyes-open resting conditions (Maestú et al., 2015; Sitnikova et al., 2018; Lopez-Martin et al., 2020; Xu et al., 2021; Yang et al., 2021; Scheijbeler et al., 2022; Vaghari et al., 2022a; Javed et al., 2025). Resting-state MEG provides relatively long segments of neural activity that enable robust estimation of oscillatory dynamics and large-scale functional connectivity, features that are particularly suitable for ML analyses.

In contrast, task-based paradigms were relatively uncommon. Only a small number of studies employed cognitive or event-related tasks. One study examined working memory processes during a cognitive task to differentiate MCI from HC (Amezquita-Sanchez et al., 2016), while another employed an auditory oddball paradigm to evaluate mismatch negativity (MMN) responses in behavioral variant frontotemporal dementia (bvFTD; Shaw et al., 2021). These findings indicate that most ML-based MEG studies in dementia research currently rely on resting-state data, with relatively limited exploration of task-evoked neural dynamics.

### MEG feature extraction

MEG features extracted across the reviewed studies spanned multiple domains of neurophysiological information, including spectral activity, functional connectivity, network dynamics, and mechanistic model parameters (Maestú et al., 2015; Koelewijn et al., 2019; Pusil et al., 2019; Shaw et al., 2021; Xu et al., 2021; Javed et al., 2025). These features reflect different aspects of neural organization and were used to capture disease-related alterations across the dementia continuum.

#### Spectral power features

Several studies focused on oscillatory spectral power within canonical frequency bands (delta, theta, alpha, beta, and gamma) at both sensor and source levels, often using beamforming or other source localization techniques (Ikeda et al., 2020; Vaghari et al., 2022a; Scheijbeler et al., 2022). Spectral power was typically computed for regions of interest (ROIs) and aggregated across subregions or the whole brain. Metrics included absolute power, relative power, normalized power, and differences between eyes-closed and eyes-open conditions.

Changes in oscillatory activity, particularly within the theta and alpha bands, were frequently associated with early-stage AD and MCI (Ikeda et al., 2020; Vaghari et al., 2022a). For instance, altered theta and alpha power in temporo-parietal regions and reduced frontal alpha activity were identified as discriminative features for early-stage AD classification (Ikeda et al., 2020). Spectral features were also used in large multi-site datasets, where sensor-level alpha and beta power measures enabled classification of MCI vs. HC with moderate accuracy (Vaghari et al., 2022a). Moreover, gamma-band (40–160 Hz) hyperactivity in right-lateralized frontal, temporal, and parietal regions was observed in young APOE ε4 carriers, highlighting the potential of high-frequency oscillations as early biomarkers of AD risk (Koelewijn et al., 2019).

#### Functional connectivity features

A substantial proportion of studies examined functional connectivity patterns derived from MEG signals (Maestú et al., 2015; Koelewijn et al., 2019; Lopez-Martin et al., 2020; Xu et al., 2021; Yang et al., 2021). Connectivity measures included amplitude envelope correlation (AEC, AEC-c), mutual information (MI), coherence, and phase coupling estimation (PCE), often corrected for signal leakage or orthogonalized between regions where appropriate (Maestú et al., 2015; Koelewijn et al., 2019; Lopez-Martin et al., 2020). Connectivity matrices were often constructed between sensors or source-reconstructed regions, producing high-dimensional representations of large-scale brain networks (Xu et al., 2021). Connectivity features were computed across multiple frequency bands (delta, theta, alpha, beta, gamma, and broadband) and aggregated across ROIs, subregions, or globally.

Across studies, connectivity measures demonstrated strong discriminatory power for distinguishing diagnostic groups (Maestú et al., 2015; Yang et al., 2021; Lopez-Martin et al., 2020). For example, hypersynchronization patterns across fronto-parietal and mid-anterior interhemispheric connections were observed in MCI patients and successfully classified individuals across multiple MEG centers (Maestú et al., 2015). Other studies reported altered connectivity patterns in parietal and temporal regions in AD, with distinct differences between early hyperconnectivity and later hypoconnectivity patterns (Koelewijn et al., 2019). Connectivity-based approaches were also applied to investigate genetic risk factors, revealing increased connectivity in young APOE ε4 carriers within alpha and beta bands in parietal and precuneus regions overlapping with the default mode network (DMN; Koelewijn et al., 2019).

#### Dynamic connectivity features and network embedding

Several studies extended connectivity analyses by examining dynamic network organization and latent network representations (Sitnikova et al., 2018; Pusil et al., 2019; Xu et al., 2021). Dynamic functional connectivity approaches capture time-varying patterns of neural interactions, enabling characterization of how functional networks fluctuate and reorganize over short timescales. In this context, some studies investigated the temporal dynamics of spontaneous neural synchrony across large-scale networks, including the DMN, dorsal attention network (DAN), visual network (VisN), sensorimotor network (SMN), and left associative network (LAN; Sitnikova et al., 2018). Temporal properties of these network states were quantified using metrics such as fractional occupancy, mean lifetime, and transition probabilities. Source-level analyses revealed short-timescale abnormalities in neural synchrony in individuals with AD, particularly within the DMN (Sitnikova et al., 2018).

Dynamic connectivity approaches have also been applied in longitudinal studies of MCI progression. Temporal fluctuations in functional connectivity were examined across multiple frequency bands and were shown to effectively distinguish stable from progressive MCI cases, with temporal decoupling between connectivity patterns assessed using dynamic time warping (DTW; Pusil et al., 2019). The most discriminative connections involved regions belonging to the DMN, cingulo-opercular (CO), fronto-parietal (FP), sensorimotor, thalamo-related, and olfactory networks, with gamma-band connectivity patterns contributing prominently to classification performance (Pusil et al., 2019).

In addition to dynamic connectivity metrics, network embedding approaches have been used to capture complex relationships within brain networks. For instance, Multiple Graph Gaussian Embedding (MG2G) generated low-dimensional representations of nodes or ROIs capturing hierarchical and non-linear properties of the connectome (Xu et al., 2021). These embedding-based features successfully differentiated neurotypical controls, stable MCI, and progressive MCI groups, highlighting their potential utility for characterizing network-level alterations across stages of cognitive decline (Xu et al., 2021).

#### Mechanistic and physiological MEG features

A smaller number of studies explored mechanistically informed neurophysiological features derived from computational modeling. For example, dynamic causal modeling (DCM) was used to estimate parameters of canonical cortical microcircuits, including layer-specific connectivity and intrinsic synaptic coupling (Shaw et al., 2021). These generative model parameters were highly informative for classifying bvFTD from HC and substantially outperformed conventional event-related response measures such as MMN amplitude (Shaw et al., 2021).

Other studies investigated markers of brain criticality and neuronal balance, including long-range temporal correlations (LRTCs) and functional excitation–inhibition ratios (fE/I) derived from resting-state MEG signals (Javed et al., 2025). These metrics captured progressive disruptions in temporal correlations and increased fE/I in individuals with progressive MCI, reflecting a shift toward supercritical brain dynamics, potentially underlying early cognitive decline and serving as sensitive biomarkers of disease progression (Javed et al., 2025).

### Integration of MEG with other neuroimaging biomarkers

Several studies explored the integration of MEG-derived features with other neuroimaging biomarkers, particularly structural neuroimaging, for anatomical reference or multimodal predictive modeling. Structural MRI was frequently used for source reconstruction and anatomical parcellation of MEG signals, enabling more precise localization of neural activity within cortical regions. In some cases, MRI features were also combined with MEG measures in multimodal classification analyses, allowing investigation of the complementary value of structural and functional brain information (Xu et al., 2021; Vaghari et al., 2022a,b).

For example, Vaghari et al. (2022b) combined MEG spectral features with MRI-derived gray matter volumes using a multiple-kernel learning framework. While MRI alone achieved 71% accuracy and MEG alone 68% in classifying MCI vs. HC, combining the modalities improved performance to 77%, highlighting the complementary information provided by functional MEG signals. Moreover, Javed et al. (2025) similarly showed that MEG-derived measures of LRTCs and fE/I were particularly informative for detecting SCD, whereas structural MRI markers, such as medial temporal lobe volumes, contributed more to distinguishing MCI.

Collectively, these studies indicate that integrating MEG with structural MRI enhances early detection of functional brain alterations and supports the use of multimodal approaches to improve classification and risk stratification.

### ML methodologies

A broad spectrum of ML algorithms was employed to analyze MEG features, reflecting the high-dimensionality of MEG data and the diversity of classification tasks. Traditional supervised learning methods were the most commonly used approaches, including SVM, RF, k-nearest neighbors (k-NN), decision trees, and logistic regression (Maestú et al., 2015; Koelewijn et al., 2019; Yang et al., 2021; Vaghari et al., 2022a). Several studies implemented ensemble learning strategies combining multiple classifiers to improve predictive robustness (Maestú et al., 2015; Scheijbeler et al., 2022). Kernel-based learning approaches were also applied to integrate multimodal datasets, allowing MEG and MRI features to be combined within a unified predictive framework (Vaghari et al., 2022b).

Another study explored deep learning architectures, particularly CNNs, to capture complex spatial relationships within connectivity matrices (Lopez-Martin et al., 2020). In this study, a randomized two-dimensional CNN ensemble (2D-CNN) achieved high classification performance for MCI detection using functional connectivity features (Lopez-Martin et al., 2020). Enhanced probabilistic neural networks (EPNN) were also implemented in one study to classify MEG-derived entropy features. Unsupervised approaches were also used in some investigations to characterize dynamic brain states, such as Hidden Markov models (HMM) applied to identify transient patterns of neural synchrony in AD (Sitnikova et al., 2018).

Across studies, cross-validation strategies (e.g., k-fold cross-validation) were commonly used to evaluate model performance and reduce the risk of overfitting when training classifiers on high-dimensional MEG feature sets.

### Prediction targets and outcomes

Prediction targets varied according to study aims, ranging from discrimination between HC and MCI, SCD, or AD, to multi-class classification of diverse dementia subtypes including DLB, FTD, and psychiatric conditions (Yang et al., 2021; Scheijbeler et al., 2022). Several studies focused on predicting conversion from MCI to AD, identifying early biomarkers of progression, or detecting preclinical changes in genetic risk carriers (Koelewijn et al., 2019; Pusil et al., 2019; Xu et al., 2021). Classification outcomes were typically reported using accuracy, AUC, sensitivity, specificity, or F1-score, and performance metrics varied according to the task complexity and features used.

### Key findings

Across the reviewed literature, several consistent neurophysiological patterns emerged. First, alterations in oscillatory brain activity, particularly within theta, alpha, and beta frequency bands, were frequently observed in individuals with cognitive impairment and AD. Specifically, MEG-based alpha and beta power measures enabled discrimination between MCI and HC with accuracies up to 68%, and integration with MRI data further improved classification to 77% (Vaghari et al., 2022a,b). Whole-brain normalization power measures in early-stage AD achieved 83% classification accuracy, with region- and frequency-specific alterations observed in theta and alpha bands, including increased theta and alpha activity in temporo-parietal regions and reduced alpha in frontal regions (Ikeda et al., 2020). These changes were often detectable even in early disease stages. Task-based analyses combining complete ensemble empirical mode decomposition, permutation entropy, and probabilistic neural networks distinguished MCI from HC during working memory tasks with 98.4% accuracy (Amezquita-Sanchez et al., 2016).

Second, functional connectivity abnormalities represented one of the most prominent findings across studies. Early stages of cognitive decline were frequently associated with hyperconnectivity or hypersynchronization in fronto-parietal and interhemispheric networks, whereas more advanced disease stages showed reduced connectivity within large-scale networks such as the DMN. Hyper-synchronization patterns in anterior-posterior and mid-anterior interhemispheric regions enabled discrimination of MCI from HC with high sensitivity but moderate specificity (Maestú et al., 2015). Studies of genetic risk carriers revealed hyperconnectivity in APOE-ε4 carriers in right-hemisphere alpha and beta bands, as well as gamma-band hyperactivity (40–160 Hz), while AD patients exhibited parieto-temporal hypoconnectivity, with SVM classifiers achieving 77.6% AUC for AD vs. HC (Koelewijn et al., 2019).

Third, dynamic and network-level features provided important insights into the temporal organization of brain activity. Disruptions in the stability and coordination of large-scale neural networks were observed in AD, reflecting altered transitions between functional brain states. Network-based embeddings captured informative functional connectivity patterns, with classification accuracies ranging from 61% to 82% for discriminating between HC, stable MCI, and progressive MCI, highlighting frontal, temporal and parietal lobe involvement (Xu et al., 2021). Dynamic functional connectivity and multiplex multi-frequency integration achieved accuracies of up to 100% for predicting conversion from MCI to AD, identifying gamma-band activity and key networks, including the DMN, CO, FP, sensorimotor, thalamo-related, and olfactory-parahippocampal networks, as critical contributors (Pusil et al., 2019). Additionally, Hidden Markov models identified short timescale abnormalities in spontaneous neural synchrony within DMN and other large-scale networks in AD patients (Sitnikova et al., 2018). Deep learning approaches, such as 2D-CNN ensembles, effectively handled high-dimensional MEG data, achieving F1-scores up to 0.92 for MCI classification (Lopez-Martin et al., 2020).

Finally, physiologically informed modeling and measures of neural criticality highlighted microcircuit-level alterations and excitation–inhibition imbalance as fundamental mechanisms underlying cognitive decline. Longitudinal analyses showed that changes in LRTCs and excitatory/inhibitory ratios predicted cognitive decline and disease progression, effectively distinguishing HC, SCD, and MCI (Javed et al., 2025). Furthermore, model-based MEG parameters, including MMN amplitude and layer-specific coupling, achieved near-perfect (99.6%) classification for bvFTD vs. HC (Shaw et al., 2021).

Regarding differential diagnosis, MEG-derived features demonstrated substantial potential for discriminating between clinical populations. Specifically, the multi-class classification of six diagnostic groups (SCD, MCI, AD, DLB, FTD, and psychiatric conditions) achieved a balanced accuracy of 41% and a multi-class AUC of 0.75, with DLB and AD exhibiting the highest sensitivities and FTD the lowest (Scheijbeler et al., 2022). Additionally, in MCI detection, source-reconstructed signals from hyper-connected ROIs achieved recognition rates up to 93.83% with robust leave-one-out cross-validation performance, while multi-resolution wavelet biomarkers further enhanced detection (Yang et al., 2021).

Taken together, these findings demonstrate that MEG-based ML approaches can capture a wide range of neurophysiological abnormalities associated with dementia and its preclinical and prodromal stages. Key biomarkers included alterations in frequency-specific oscillatory activity, functional connectivity patterns, and large-scale network dynamics, while multimodal approaches integrating MEG with structural neuroimaging such as MRI further improved predictive performance. Reported classification accuracies ranged from modest (~60%) to near-perfect performance (100%) in targeted cohorts, with most studies exceeding 80% for distinguishing diagnostic categories or predicting MCI-to-AD progression. For more detailed information regarding the performance metrics of each study and model, please refer to summary Table 1.

## Discussion

### Overview and current evidence

This scoping review synthesized current evidence on AI–driven analysis of MEG data for the classification, prediction, and prognosis of MCI and dementia. Fourteen studies published within the past decade met the eligibility criteria, reflecting the growing interest in combining advanced computational techniques with high-temporal-resolution neurophysiological recordings. Although the number of studies remains relatively limited, the findings collectively highlight the potential of AI-driven MEG analysis to detect subtle functional alterations associated with early neurodegenerative processes.

Across the reviewed studies, spectral features—particularly theta, alpha and beta power—were consistently informative for distinguishing MCI and early dementia from healthy aging. These features likely perform well because oscillatory activity reflects fundamental aspects of cortical excitability, synaptic communication, and excitation–inhibition balance, which are among the first neurophysiological processes affected in neurodegenerative disease (Ikeda et al., 2020; Javed et al., 2025). Spectral measures also tend to have relatively high signal-to-noise ratios at the sensor level, making them robust across recording sites and participants, and they can capture subtle alterations even before overt cognitive symptoms appear.

However, functional connectivity and network-based metrics provided even greater sensitivity, particularly for early-stage or preclinical populations. Connectivity analyses quantify interactions between brain regions, capturing distributed network disruptions that may precede measurable spectral changes or structural atrophy (Koelewijn et al., 2019; Pusil et al., 2019). For example, in cognitively healthy APOE ε4 carriers, subtle hyperconnectivity in fronto-parietal and DMN has been observed years before symptom onset, suggesting that network-level disturbances may serve as early biomarkers of latent neurodegeneration (Koelewijn et al., 2019). These findings may suggest the ability of connectivity metrics to reflect compensatory mechanisms, regional vulnerability, and progressive breakdown of network integration—features that are directly relevant to the cognitive domains affected in MCI and dementia.

Another important insight emerging from the reviewed literature concerns the dynamic nature of these network alterations. Specifically, early stages of cognitive impairment appear to involve increased synchronization or hyperconnectivity across specific brain regions and frequency bands, likely reflecting compensatory neural mechanisms to maintain cognitive function (Maestú et al., 2015; Yang et al., 2021). As neurodegeneration progresses, however, this initial hyper-synchronization shifts toward reduced connectivity, diminished oscillatory coordination, and widespread disruption of large-scale network integration (Koelewijn et al., 2019; Sitnikova et al., 2018). This transition from hyper- to hypo-synchronization illustrates the non-linear trajectory of cortical reorganization during dementia progression and suggests that disease evolution may be better conceptualized as a gradual loss of network flexibility and communication efficiency rather than abrupt structural disconnections (Xu et al., 2021).

Taken together, spectral features provide a reliable measure of local neuronal activity and excitability, while connectivity and network-based measures capture the coordinated dynamics of distributed brain systems. The complementary nature of these features suggests that multimodal analyses combining spectral and connectivity measures may yield superior classification performance, as they integrate both types of information.

AI-driven analytical frameworks appear particularly well suited to capture these multidimensional patterns of neural dysfunction in neurodegeneration. By integrating spectral features, connectivity metrics, graph-theoretical network properties, and measures of neural complexity, ML approaches can reveal distributed electrophysiological signatures that extend beyond traditional univariate analyses (Vaghari et al., 2022b; Javed et al., 2025). Several studies further demonstrated that AI approaches can identify subtle temporal interactions and evolving network patterns, offering predictive insights into disease progression at early stages (Pusil et al., 2019; Xu et al., 2021).

Taken together, the current evidence suggests that AI-driven analysis of MEG data captures multidimensional signatures of neurodegeneration encompassing oscillatory slowing, altered cortical activity, reduced signal complexity, connectivity disturbances, and progressive network instability, yielding consistently moderate to high classification performance across studies. Importantly, the observations of the present study align with broader evidence from neurophysiological studies of aging. A recent comprehensive systematic review by Fernández-Rubio and colleagues, examining more than 900 EEG and MEG studies of healthy and pathological aging, identified several recurrent electrophysiological markers of cognitive decline, including spectral slowing, reductions in signal complexity, decreased coherence, and attenuated event-related responses (Fernández-Rubio et al., 2025).

These findings support the growing role of MEG as a sensitive, non-invasive tool for detecting early functional brain alterations associated with dementia, complementing traditional structural and molecular biomarkers (Vaghari et al., 2022b).

### Clinical implications

The findings summarized in this review highlight several important clinical implications. Because electrophysiological alterations may emerge before detectable structural changes, MEG provides a unique opportunity to identify early functional signatures of neurodegenerative processes (Vaghari et al., 2022b; Javed et al., 2025). AI-driven analysis may therefore complement existing diagnostic frameworks by providing objective and non-invasive measures of functional brain integrity.

A particularly relevant clinical application involves predicting progression from MCI to dementia. Determining which individuals with MCI will remain stable and which will develop dementia is critical for patient counseling, care planning, and the timely initiation of therapeutic interventions (Pusil et al., 2019; Xu et al., 2021; Javed et al., 2025). The reviewed studies suggest that MEG-derived biomarkers—particularly those reflecting oscillatory dynamics and network-level connectivity—may capture early neurophysiological changes associated with disease progression, supporting the possibility of individualized risk stratification during the preclinical and prodromal stages of neurodegeneration (Koelewijn et al., 2019; Maestú et al., 2015). ML-assisted MEG analysis may also contribute to improved differentiation between dementia subtypes. Conditions such as AD, FTD, DLB, and vascular dementia often present with overlapping clinical symptoms but involve distinct patterns of neural network disruption. Improved electrophysiological characterization of these patterns could therefore facilitate more accurate diagnosis and support the development of targeted therapeutic strategies.

### Methodological challenges and research gaps

Despite encouraging progress, several methodological challenges emerged in the reviewed studies, highlighting factors that continue to limit the clinical translation of AI-driven MEG research. Many of these challenges mirror broader limitations identified in large-scale neurophysiological investigations of aging (Fernández-Rubio et al., 2025).

First, a prominent challenge concerns the considerable heterogeneity across studies in terms of participant populations, recording protocols, preprocessing strategies, feature extraction methods, and machine learning algorithms. This variability complicates direct comparisons between studies and makes it difficult to determine which biomarkers are most robust. Similar issues have been noted in large reviews of neurophysiological research on aging, where the proliferation of diverse analytical metrics has often hindered the development of unified frameworks for understanding dementia-related changes in brain activity (Fernández-Rubio et al., 2025).

Another limitation is the predominance of cross-sectional designs. Among the reviewed studies, only a few employed longitudinal research design, and even these had relatively short observation periods, often less than 3 years. Given that neurodegenerative diseases evolve gradually over many years, the scarcity of long-term longitudinal data limits the ability to determine whether identified electrophysiological markers can reliably predict individual disease trajectories (Fernández-Rubio et al., 2025; Pusil et al., 2019).

Sample size is also a consistent constraint. Many studies included relatively small cohorts, increasing the risk of model overfitting and reducing the generalizability of predictive algorithms, particularly when complex ML and deep learning approaches were applied to high-dimensional MEG data (Amezquita-Sanchez et al., 2016; Sitnikova et al., 2018).

Another methodological pattern concerns the widespread reliance on resting-state recordings, often obtained under eyes-closed conditions. While these paradigms are straightforward to implement and facilitate comparison across studies, they capture only a limited aspect of cognitive brain function. In this review, only a minority of studies incorporated task-based paradigms, and the consistency of findings across resting and task-based conditions remains underexplored (Amezquita-Sanchez et al., 2016; Shaw et al., 2021).

Finally, most of the included studies focused on identifying biomarkers that differentiate diagnostic groups at the population level. While such approaches have yielded valuable insights into disease mechanisms, their direct clinical applicability remains limited. Few studies have validated biomarkers at the level of individual participants, which is essential for developing tools suitable for personalized risk prediction and clinical decision-making (Pusil et al., 2019; Fernández-Rubio et al., 2025).

In summary, the reviewed studies demonstrate that heterogeneity in methods, limited longitudinal data, small sample sizes, overreliance on resting-state recordings, and a predominant focus on group-level analyses collectively pose significant challenges to advancing AI-driven MEG research toward clinical application.

### Recommendations for future research

Addressing the methodological challenges identified in the reviewed studies will require coordinated methodological, technical, and collaborative efforts across the field. A key priority is the development of longitudinal studies incorporating repeated neurophysiological recordings over extended periods. Given the gradual progression of dementia, longer follow-up windows—ideally 5–10 years—are necessary to capture transitions from healthy aging to MCI and dementia and to validate the predictive value of electrophysiological biomarkers (Pusil et al., 2019; García-Colomo et al., 2024; Fernández-Rubio et al., 2025).

Expanding the range of experimental paradigms in MEG research represents another important direction (Fernández-Rubio et al., 2025). While resting-state recordings provide valuable baseline measures, task-based paradigms probing memory, attention, executive functioning, and predictive processing may reveal additional neural alterations relevant to cognitive decline. Developing standardized task protocols that can be applied across research centers would improve comparability, reduce methodological heterogeneity, and enhance the translational value of findings.

Another critical priority involves the creation of large multicenter datasets and the expansion of open data-sharing initiatives (Fernández-Rubio et al., 2025). Collaborative databases integrating neurophysiological recordings from multiple sites could significantly increase statistical power and enable the development of more robust ML models. Such efforts may also help address the geographical imbalance currently observed in neurophysiology research, which remains disproportionately concentrated in high-income countries despite the global burden of dementia.

Future studies should also increasingly integrate neurophysiological measures with other established biomarkers of neurodegeneration, within a broader diagnostic framework. Combining MEG-derived features with structural neuroimaging, molecular biomarkers, genetic information, and neuropsychological assessments may provide a more comprehensive characterization of disease mechanisms and improve predictive accuracy (Vaghari et al., 2022b; Chatzidimitriou et al., 2025). Multimodal approaches may also support individualized risk stratification and early detection of disease progression.

Importantly, future work should also prioritize the development of biomarkers that can be applied at the level of individual patients. While group-level analyses are valuable for identifying disease mechanisms, clinical implementation ultimately requires tools capable of evaluating individual brain function and predicting personalized risk trajectories (Fernández-Rubio et al., 2025). Achieving this goal will require standardized experimental paradigms, reproducible analytical pipelines, and validation across large longitudinal cohorts (García-Colomo et al., 2024).

Finally, the integration of explainable AI approaches is essential for clinical translation. Models capable of highlighting the neural features contributing to predictions—such as alterations in specific frequency bands or connectivity networks—can enhance interpretability and improve clinician confidence in AI-assisted decision-making tools (Rossini et al., 2022). Alongside this, transparent reporting and consistent standards are crucial for moving AI-MEG research from experimental studies to reliable clinical applications. Following established frameworks, such as the FAIR data principles and reporting guidelines like TRIPOD-AI, CLAIM-2024, SPIRIT-AI, and CONSORT-AI, helps ensure transparency in how models are designed, trained, and tested. These initiatives encourage thorough documentation of data sources, feature extraction, and analytical steps, which improves comparability between studies and supports external validation. Embedding such standards into future MEG–AI research will bridge technical innovation with clinical practice, strengthening trust, reproducibility, and safety in the use of predictive neurotechnologies.

### Emerging MEG and AI methodologies

Recent methodological advances in MEG technology have created exciting opportunities to enhance the spatial and temporal precision in neurophysiological measurements. Notably, OPMs have emerged as a transformative technology capable of addressing several limitations of traditional cryogenic MEG systems. OPM-MEG allows for wearable, flexible, and closer-to-scalp recordings, resulting in higher signal-to-noise ratios, improved comfort, and greater tolerance for participant movement. Unlike cryogenic systems that rely on liquid helium cooling and fixed head positioning, OPM-based systems are portable and cost-effective, enabling more naturalistic paradigms such as ambulatory monitoring or task-based interaction (Brookes et al., 2022; Boto et al., 2018).

At the same time, advances in ML are transforming how neurophysiological data are analyzed. Traditional analytical pipelines typically rely on predefined feature extraction steps, in which researchers manually compute spectral or connectivity metrics before applying ML algorithms. More recent developments have introduced end-to-end learning frameworks, such as Braindecode (Heilmeyer et al., 2018), capable of operating directly on minimally processed electrophysiological signals (Kostas et al., 2019). In these architectures, neural networks learn hierarchical representations of brain activity directly from the raw data, automatically extracting spatial, temporal, and spectral patterns relevant to the prediction task. Such approaches can capture complex dependencies within MEG signals—including interactions between sensors, temporal dynamics across frequency bands, and non-linear patterns of neural activity—that may be difficult to model using conventional feature-based methods.

Another emerging direction involves the development of large-scale foundation models trained on extensive electrophysiological datasets (Kwon and Shin, 2025). Inspired by advances in natural language processing (NLP) and computer vision, these models aim to learn generalizable representations of brain activity that can subsequently be adapted to specific tasks through transfer learning. In practice, a foundation model can be trained on large datasets of EEG or MEG recordings to learn fundamental patterns of neural activity, and then fine-tuned for specialized applications such as disease classification, cognitive state decoding, or prediction of disease progression. Although still in early stages, such approaches may help address key challenges currently faced by neurophysiological ML research, including limited dataset sizes, heterogeneity across studies, and the difficulty of training robust models on small clinical cohorts.

### Limitations of this review

Despite its contributions, this review has several limitations. The literature search was conducted exclusively in PubMed (MEDLINE), potentially omitting relevant studies indexed in other databases such as Scopus, Embase, IEEE Xplore, or Web of Science. The focus on studies published between 2015 and 2025 to capture recent methodological developments may have excluded earlier foundational research. Only English-language publications were considered, introducing potential language bias. Furthermore, the review employed a qualitative synthesis rather than a quantitative meta-analysis, limiting the ability to statistically compare performance metrics across studies or to assess publication bias. Consequently, while this scoping review provides a valuable overview of trends, methodological approaches, and gaps in AI-driven MEG dementia research, future systematic reviews and meta-analyses are warranted to quantitatively evaluate diagnostic accuracy, effect sizes, and methodological quality across the literature.

### Conclusions

AI-driven analysis of MEG data demonstrates substantial potential for improving the early detection and prognosis of cognitive decline and dementia. Despite the limited number of studies, current evidence suggests that MEG features—including oscillatory activity, functional connectivity patterns, neural complexity measures, and large-scale network dynamics—especially when combined with multimodal data, can discriminate between clinical populations with moderate to high accuracy and classification performance. Nonetheless, challenges related to small sample sizes, methodological heterogeneity, and limited model interpretability remain significant barriers impeding clinical translation.

To advance the field, future efforts should emphasize standardized preprocessing pipelines, multicenter collaborations, and longitudinal study designs, alongside explainable AI frameworks that foster clinical transparency and trust. Emerging MEG technologies, such as OPM-MEG, together with advanced AI approaches—including end-to-end learning frameworks and large-scale foundation models—can integrate multimodal biomarkers (e.g., MRI, PET, genetics, neuropsychological assessments, and clinical data) to improve individualized risk prediction, refine prognostic models, and guide precision therapeutic strategies. As methodological rigor and interdisciplinary collaboration continue to strengthen, AI-driven MEG analysis holds strong promise to become a transformative tool for the early diagnosis and personalized management of dementia.
